# Inhibition of glycine transporter-1 in the dorsal vagal complex improves metabolic homeostasis in diabetes and obesity

**DOI:** 10.1038/ncomms13501

**Published:** 2016-11-22

**Authors:** Jessica T. Y. Yue, Mona A. Abraham, Paige V. Bauer, Mary P. LaPierre, Peili Wang, Frank A. Duca, Beatrice M. Filippi, Owen Chan, Tony K. T. Lam

**Affiliations:** 1 Toronto General Hospital Research Institute and Department of Medicine, UHN, Toronto, Ontario, Canada M5G 1L7; 2 Departments of Physiology, Toronto, Ontario, Canada M5S 1A8; 3 Department of Internal Medicine, Section of Endocrinology, Yale University School of Medicine, New Haven, Connecticut 06520, USA; 4 Departments of Medicine, University of Toronto, Toronto, Ontario, Canada M5S 1A8; 5 Banting and Best Diabetes Centre, University of Toronto, Toronto, Ontario, Canada M5G 2C4

## Abstract

Impaired glucose homeostasis and energy balance are integral to the pathophysiology of diabetes and obesity. Here we show that administration of a glycine transporter 1 (GlyT1) inhibitor, or molecular GlyT1 knockdown, in the dorsal vagal complex (DVC) suppresses glucose production, increases glucose tolerance and reduces food intake and body weight gain in healthy, obese and diabetic rats. These findings provide proof of concept that GlyT1 inhibition in the brain improves glucose and energy homeostasis. Considering the clinical safety and efficacy of GlyT1 inhibitors in raising glycine levels in clinical trials for schizophrenia, we propose that GlyT1 inhibitors have the potential to be repurposed as a treatment of both obesity and diabetes.

## Abstract (web summary)

Glycine sensing in the dorsal vagal complex (DVC) regulates hepatic glucose production in rodents. Here the authors show that pharmacological and molecular inhibition of glycine reuptake in the DVC potentiates NMDA receptors, and improves metabolic homeostasis in animal models of obesity and diabetes.

## Graphical abstract

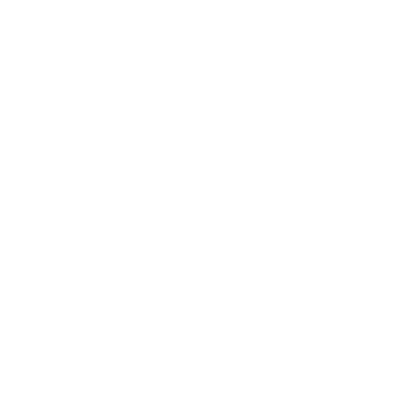

Obesity and diabetes have become a worldwide epidemic. Over 2.1 billion people worldwide are overweight or obese^1^ and approximately 422 million are afflicted with diabetes^2^. Given the combined economic burden of treating both these diseases and their complications, the development of safe and effective therapeutic strategies is decidedly crucial. The dysregulation of glucose and energy homeostasis in diabetes and obesity are caused in part by the aberrant elevation of hepatic glucose production and energy intake^3^,^4^, pathologies which arise from the collective failure of multiple homeostatic systems involving the liver, pancreas, adipose tissue, brain and gastrointestinal tract^4^,^5^,^6^,^7^,^8^. As such, the development of pharmacological approaches to restore the impaired mechanisms within these systems is crucial to restore metabolic homeostasis in diabetes and obesity. Neural circuits of the central nervous system (CNS) emerge as a potential target for clinical intervention. The recently FDA-approved anti-obesity drug, lorcaserin, activates hypothalamic 5-HT_2C_ receptors to reduce food intake^9^. Moreover, the anti-diabetic glucagon-like-peptide 1 receptor agonist drug, liraglutide, which is clinically demonstrated to improve glycemia and reduce body weight^10^, requires activation of neuronal glucagon-like-peptide 1 receptor to exert its anorectic effects^11^. The central actions of other hormones such as insulin further demonstrate the potential of CNS-based therapies, as intranasal insulin delivery in humans reduces food intake^12^ and glucose production^13^ and improves whole-body insulin sensitivity^14^.

CNS nutrient sensing mechanisms also reduce food intake and body weight^15^ and lower glucose levels in healthy^16^ and diabetic^17^ rodents. Specifically, hypothalamic nutrient sensing activates a forebrain–hindbrain neuronal axis involving *N*-methyl-d-aspartate (NMDA) receptors in the dorsal vagal complex (DVC) to suppress glucose production^18^, while these same DVC NMDA receptors are required for intestinal sensing of nutrients, as well as metformin and resveratrol, to lower glucose production and food intake^5^,^19^,^20^,^21^,^22^. Furthermore, directly targeting the DVC has metabolic benefits, as direct administration of glycine, an obligatory co-agonist of the NMDA receptor, into the DVC of healthy rats lowers glucose production via NMDA receptor activation^23^. Therefore, manipulating glycine levels in the DVC could present a therapeutic target for the treatment of obesity and diabetes. However, administration of glycine *per se* is not suitable as a therapy due to its poor pharmacokinetics *in vivo*.

On the other hand, regulating glycine concentration by manipulating glycine transporters (GlyT) has demonstrated clinical feasibility. Since glycine uptake into cells is regulated by glycine transporters, of which GlyT1 is the primary regulator of glycine levels in the vicinity of NMDA receptors^24^, GlyT1 inhibition therefore increases extracellular glycine levels to potentiate the activation of NMDA receptors^25^. Modulation of NMDA receptor neurotransmission is currently used as a therapy for schizophrenia, a disease that displays reduced NMDA receptor function. In fact, the clinical trials have shown that augmentation of NMDA receptor function via GlyT1 inhibitors improve symptoms of schizophrenia^26^,^27^. However, no studies to date have investigated the therapeutic potential of GlyT1 inhibition for the treatment of diabetes and obesity. Here, we examined whether GlyT1 inhibition regulates glucose and energy homeostasis in healthy, obese and diabetic rodents (Fig. 1a). We demonstrate that direct inhibition of GlyT1 in the DVC confers metabolic benefits including improved glucose tolerance, lowered glucose production, reduced feeding and lowered body weight gain in diabetic and obese rodents. We also report that systemic infusion of GlyT1 inhibitor recapitulates the metabolic effects of DVC GlyT1 inhibition. Thus, inhibiting GlyT1 in the brain represents a potential novel therapeutic strategy to lower plasma glucose levels and body weight in diabetes and obesity.

## Results

### Gluco-regulation by DVC GlyT1 inhibition in healthy rodents

**Figure 1:**
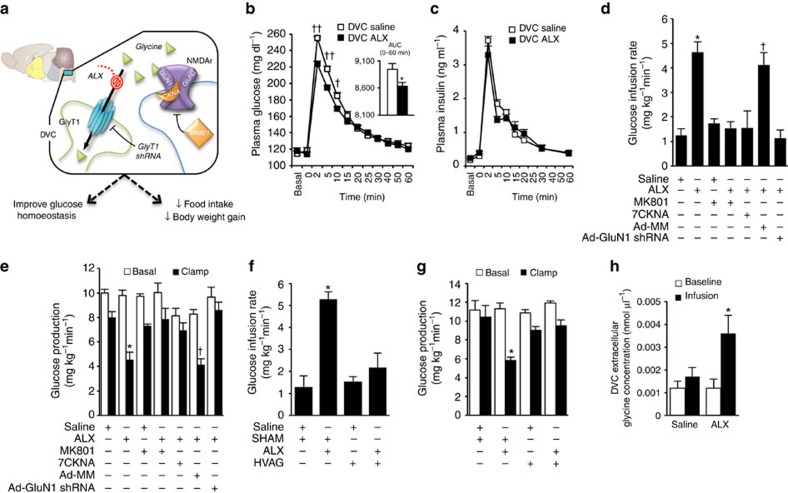
Chemical inhibition of DVC GlyT1 regulates glucose homeostasis in healthy rats. (**a**) Schematic representation of working hypothesis: glycine transporter-1 (GlyT1) facilitates the cellular uptake of glycine in the dorsal vagal complex (DVC). Chemical (via DVC ALX infusion) or genetic (via DVC lentiviral injection of GlyT1 shRNA) inhibition of GlyT1 increases extracellular glycine levels in the DVC, which potentiates the activation of DVC *N*-methyl-D-aspartate receptors (NMDAr) to regulate glucose production and glucose tolerance, and food intake and body weight gain. MK-801, NMDAr ion channel blocker; 7-chlorokynurenic acid, 7CKNA-antagonist to the GluN1 subunit of NMDAr. (**b**) Plasma glucose levels (inset: integrated area under the curve (AUC)) and (**c**) plasma insulin levels during ivGTT with DVC infusion of ALX (*n*=8, black squares) or saline (*n*=7, white squares). ^†^*P*<0.04, ^††^*P*<0.0008 determined by Sidak's multiple comparisons test following repeated-measures ANOVA. *P<0.05 determined by *t*-test. (**d**) Glucose infusion rates and (**e**) glucose production during clamps with DVC infusion of saline (*n*=11), ALX (*n*=9), MK801 (*n*=9), ALX+MK801 (*n*=5), ALX+7CKNA (*n*=5), Ad-MM+ALX (*n*=5) or Ad-GluN1 shRNA+ALX (*n*=5). (**d**: *P<0.002 versus saline, MK801, ALX+MK801, and ALX+7CKNA determined by ANOVA and Dunnett's *post hoc* test; ^†^*P*<0.002 versus Ad-GluN1 shRNA+ALX determined by *t*-test; (**e**) *P<0.02 versus saline, MK801, ALX+MK801 and ALX+7CKNA determined by ANOVA and Dunnett's *post hoc* test; ^†^*P*<0.0008 versus Ad-GluN1 shRNA+ALX determined by *t*-test.) (**f**) Glucose infusion rates and (**g**) glucose production during clamps with DVC ALX infusion in vagotomized (*n*=7) or sham-operated (*n*=5) rats or DVC saline infusion in vagotomized (*n*=7) or sham-operated rats (*n*=5) rats. (for **f** and **g** *P<0.01 compared with all the other groups determined by ANOVA and Dunnet's *post hoc* test). (**h**) Extracellular glycine levels within the DVC following DVC infusion of ALX (*n*=7) or saline (*n*=7) in microdialysis studies. *P<0.03 versus saline determined by *t-*test. Data are shown as the mean+s.e.m.

To first assess a gluco-regulatory function of GlyT1 inhibition in physiological conditions, we infused the GlyT1 inhibitor, ALX^28^,^29^, into the DVC of conscious, unrestrained healthy rats and monitored plasma glucose levels during an intravenous glucose tolerance test (ivGTT; Supplementary Fig. 1a). DVC ALX infusion for 5 h is found to improve glucose tolerance (Fig. 1b) independent of a rise in plasma insulin levels (Fig. 1c) compared with DVC saline infusions. To begin delineating the mechanism by which DVC GlyT1 inhibition improves glucose tolerance independent of changes in insulin, we tested whether DVC GlyT1 inhibition regulates glucose production or uptake during pancreatic basal insulin euglycaemic clamps in both rats and mice (Supplementary Fig. 1a), since DVC glycine infusion potentiates NMDA receptors to inhibit hepatic glucose production^23^.

Infusion of ALX into the DVC of rats increases the requirement for exogenous glucose infusion to maintain euglycaemia (Fig. 1d) and lowers the rate of glucose production (Fig. 1e) compared with infusions of saline, independent of differences in glucose uptake, plasma glucose, plasma insulin or body weight (Supplementary Fig. 1c–f). We also performed pancreatic clamps in healthy mice that underwent stereotaxic and vascular surgeries and demonstrated that ICV-fourth ventricle ALX infusion correspondingly increases glucose infusion rates and suppresses glucose production without affecting glucose uptake or plasma glucose levels (Supplementary Fig. 2a–d).

Although inhibition of NMDA receptors with DVC infusion of NMDA receptor blocker MK801 alone has no effect on glucose metabolism in rats, the ability of DVC ALX infusion to increase glucose infusion rates and suppress glucose production is abolished with co-infusion of MK801 (Fig. 1d,e), without altering glucose uptake or plasma glucose (Supplementary Fig. 1c,d). The classical NMDA receptors comprised of two glycine-binding GluN1 subunits and two glutamate-binding GluN2 subunits require co-agonism of their subunit binding sites for activation^25^,^30^,^31^,^32^. Since GluN1 is an obligatory subunit^31^ and full agonism at the glycine site is necessary for full NMDA receptor activation^33^, we tested the gluco-regulatory ability of DVC ALX infusion when GluN1 is inactivated. Similar to that which was observed with NMDA receptor inhibition, specific chemical antagonism of the GluN1 subunit of NMDA receptors with 7-chlorokynurenic acid (7CKNA) into the DVC nullifies the ALX-induced increase of the requirement for exogenous glucose and suppression of glucose production without affecting glucose uptake or plasma glucose levels (Fig. 1d,e. Supplementary Fig. 1c,d). Selective genetic inhibition of DVC GluN1 subunits with injection of an adenoviral vector expressing GluN1 short hairpin RNA (Ad-GluN1 shRNA) likewise reverses the ability of ALX infusion to increase glucose infusion rates and lower glucose production compared with adenovirus-injected mismatch sequence (Ad-MM) controls (Fig. 1d,e, Supplementary Fig. 1c,d).

To test whether hepatic vagal innervation mediates the gluco-regulatory effects of DVC GlyT1 inhibition, we examined the effect of DVC ALX in rats with hepatic vagotomy versus sham surgery. While hepatic vagotomy or sham surgery *per se* do not affect glucose kinetics, the higher glucose infusion rate and lower glucose production observed in sham rats receiving DVC ALX compared with DVC saline are negated in hepatic vagotomized rats, without any difference in glucose uptake or plasma glucose (Fig. 1f,g, Supplementary Fig. 2e,f).

We next performed microdialysis to examine the effect ALX infused into the DVC would have on the extracellular levels of glycine within the DVC in healthy rats *in vivo* (Supplementary Fig. 1b). When ALX versus saline is infused into the DVC at a comparable duration and dosage as the ivGTT and clamp infusion studies in healthy rats (Supplementary Fig. 1a), ALX versus saline results in a ∼2.5-fold increase in extracellular glycine levels in the DVC (Fig. 1h). Taken together, DVC GlyT1 inhibition via ALX infusion increases glucose tolerance and elevates extracellular glycine levels in the DVC to potentiate NMDA receptors and activate a brain–liver axis to lower glucose production in healthy rodents *in vivo*.

**Figure 2:**
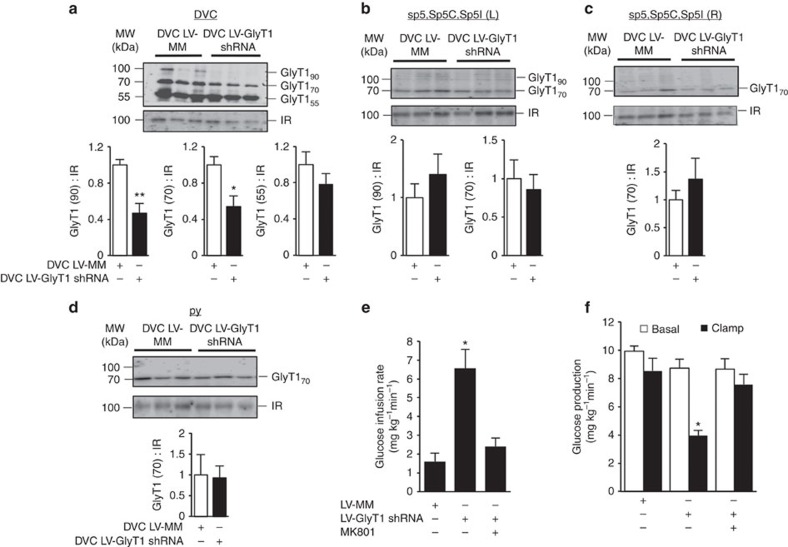
Molecular inhibition of DVC GlyT1 regulates glucose homeostasis in healthy rats. (**a**) Representative western blots and protein levels of plasma membrane GlyT1 (55, 70 and 90 kDa isoforms) normalized to insulin receptor (IR) in DVC wedges of rats 13-day post DVC lentiviral (LV) injection of GlyT1 shRNA (black bars, *n*=14) or a mismatch sequence (MM; white bars, *n*=11) as a control. *P<0.01, **P<0.001 determined by *t*-test. (**b**–**d**) Representative western blots and protein levels of plasma membrane GlyT1 (70 and/or 90 kDa isoforms) normalized to IR in sp5, Sp5C, Sp5I (L), sp5, Sp5C, Sp5I (R) and py wedges of rats 13-day post DVC LV injection of GlyT1 shRNA (black bars, *n*=5) or MM (white bars, *n*=5). (**e**) Glucose infusion rates and (**f**) glucose production during clamps in rats injected with LV-MM (*n*=7), LV-GlyT1 shRNA (*n*=7) or LV-GlyT1 shRNA with DVC MK801 infusion (*n*=6). (**e**: *P<0.006; **f**: *P<0.003 versus LV-MM control and LV-GlyT1 shRNA+MK801 determined by ANOVA and Dunnett's *post hoc* test). Data are shown as the mean+s.e.m.

We alternatively tested the gluco-regulatory role of hindbrain GlyT1 inhibition via the targeted molecular knockdown of GlyT1 within the DVC. We first confirmed that lentiviral injection of GlyT1 shRNA (LV-GlyT1 shRNA) into the DVC selectively reduces the expression of both the 70- and 90-kDa isoforms of GlyT1 in plasma membrane fractions of only the DVC tissue compared with lentiviral injection of mismatch sequence (LV-MM), but not in the two adjacent left and right lateral regions of the DVC containing the Spinal trigeminal track (sp5), Spinal 5nu caudal part (Sp5C) and Spinal 5nu interpolar (Sp5I), and the region inferior to the DVC containing the pyramidal tract (py) of the same rats (Fig. 2a–d, Supplementary Fig. 3i–v). The dominant band at the molecular weight of 70–75 kDa corresponds to GlyT1a and b isoforms and the weaker band at 90–100 kDa corresponds to GlyT1c isoform found in the rat brain as described^34^. The 90–100 kDa band is not detected in the sp5, Sp5C, Sp5I (right) and py regions (Fig. 2c,d). The immunoblot also reveals a strong band at 55 kDa (Fig. 2a), which is consistent with the occurrence of the partially glycosylated form of GlyT1 in the 55–60 kDa range as indicated^35^,^36^,^37^. However, the 55 kDa GlyT1 band in the DVC of LV-GlyT1 shRNA versus LV-MM injected rats is not significantly different as compared with the effect on 70 kDa and 90 kDa bands (Fig. 2a). Nonetheless, the specific metabolic role of various forms of GlyT1 in the brain warrants future investigation.

A 13-day chronic inhibition of GlyT1 robustly increases glucose infusion rates (Fig. 2e) and diminishes rates of glucose production (Fig. 2f) during the clamps, independent of changes in glucose uptake and plasma glucose (Supplementary Fig. 4a,b). The DVC LV-GlyT1 shRNA and -MM injected regular-chow-fed rats received vascular surgery (for the clamp studies) on day 8 post DVC viral injection and the clamp studies were conducted on day 13 (Supplementary Fig. 1a). The body weights of these viral injected rats remain comparable on the morning of the clamps at which point the rats were also fasted for 4–6 h (Supplementary Fig. 4c). Importantly, the gluco-suppressive effect of this chronic molecular GlyT1 inhibition is also mediated through the activation of DVC NMDA receptors since DVC infusion with MK801 abolishes the effect of LV-GlyT1 shRNA to increase glucose infusion rates and lower glucose production, unaffected by differences in glucose uptake, glycaemia or body weight (Fig. 2e,f, Supplementary Fig. 4a–c). These molecular loss-of-function studies strengthen the role of DVC GlyT1 inhibition in elevating extracellular glycine levels and activating NMDA receptors to lower glucose production in healthy rodents.

### Anti-diabetic effect of DVC GlyT1 inhibition

**Figure 3:**
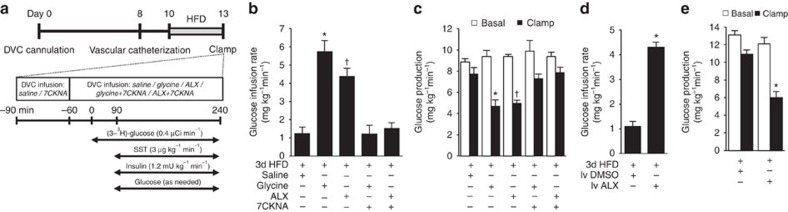
DVC and intravenous infusion of ALX regulates glucose homeostasis in 3d-HFD rats. (**a**) Experimental protocol for **b**–**c**. (**b**) Glucose infusion rates and (**c**) glucose production during clamps with DVC infusion of saline (*n*=5), glycine (*n*=6), ALX (*n*=5), glycine+7CKNA (*n*=5) and ALX+7CKNA (*n*=5). (**b**: *P<0.0003 versus saline and glycine+7CKNA; ^†^*P*<0.001 versus saline and ALX+7CKNA; determined by ANOVA and Dunnett's *post hoc* test; **c**: *P<0.006 versus saline and glycine+7CKNA; ^†^*P*<0.002 versus saline and ALX+7CKNA; determined by ANOVA and Dunnett's *post hoc* test.) (**d**) Glucose infusion rates and (**e**) glucose production during clamps with intravenous infusion of 6% DMSO (*n*=7) or ALX (*n*=7) in 3-d HFD rats. (**d**,**e**: *P<0.001 versus intravenous DMSO determined by *t*-test). Data are shown as the mean + s.e.m.

We next sought to ascertain a therapeutic relevance for the glucose-lowering capacity of DVC GlyT1 inhibition first in 3-day high-fat-diet (3-d HFD)-fed rats (Fig. 3a). The rats placed on a 3-d HFD were first confirmed to be hyperphagic (cumulative food intake: 258±10 versus 178±11 kcal, *P*<0.01 3-d HFD (*n*=26) versus 3-d RC (*n*=11), *t*-tests) and hyperinsulinemic (3-d HFD (1.7±0.2, *n*=5) versus 3d RC rats (0.9±0.1, *n*=8), *P*<0.05, *t-*tests), consistent with the fact that 3d HFD rats were validated in parallel under hyperinsulinemic–euglycaemic clamp conditions in our research facility to exhibit hepatic insulin resistance^20^. We here evaluated whether antagonism of DVC GlyT1 modulates glucose homeostasis in these 3-d HFD rats to the same extent as direct DVC glycine infusion during the pancreatic (basal insulin)–euglycaemic clamp conditions, given that DVC GlyT1 inhibition increases extracellular DVC glycine levels (Fig. 1h). Indeed, DVC GlyT1 inhibition with ALX increases the requirement of glucose (Fig. 3b) and suppresses the rate of glucose production (Fig. 3c) independent of alterations in glucose uptake (Supplementary Fig. 5a) and plasma glucose levels (Supplementary Fig. 5b) during the pancreatic clamp in HFD rats to the same extent as DVC glycine infusion (Fig. 3b,c). Further, this glucose production-lowering effect of DVC ALX or glycine in 3-d HFD rats requires the activation of the NMDA receptor GluN1 subunits as co-infusion of 7CKNA with ALX or glycine abates the glucose-suppressive ability of ALX and glycine (Fig. 3b,c).

We next examined whether systemic administration of GlyT1 inhibitor ALX recapitulates the glucose production-lowering effect of DVC ALX infused-dependent GlyT1 inhibition in 3-d HFD-fed rats. Strikingly, constant intravenous infusion of ALX for 5 h leads to a higher glucose infusion rate (Fig. 3d) and lower glucose production (Fig. 3e) compared with intravenous 6% dimethyl sulfate (DMSO) vehicle infusion during pancreatic clamps, and these metabolic changes occur independent of glucose uptake (Supplementary Fig. 5c) and changes in plasma glucose levels (Supplementary Fig. 5d).

**Figure 4:**
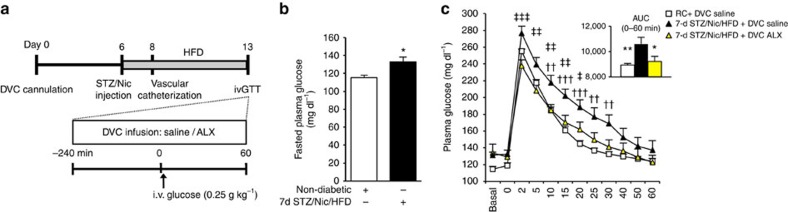
Inhibition of DVC GlyT1 regulates glucose homeostasis in diabetic rats. (**a**) Experimental protocol for **b**–**c**. (**b**) Plasma levels of glucose in overnight-fasted 7d STZ/Nic/HFD diabetic rats (black bars, *n*=17) compared with non-diabetic, regular chow-fed counterparts (white bars, *n*=13); *P<0.01 determined by *t*-test. (**c**) Plasma glucose levels (inset: integrated area under the curve (AUC)) during ivGTT with DVC infusion of ALX (*n*=9, grey triangles) or saline (*n*=8, black triangles) in 7d STZ/Nic/HFD rats or DVC saline in regular chow rats (*n*=7, white squares). ^††^*P*<0.01, ^†††^*P*<0.001 versus DVC saline+regular chow rats; ^‡^*P*<0.05, ^‡‡^*P*<0.01, ^‡‡‡^*P*<0.001 versus DVC ALX+7d STZ/Nic/HFD rats determined by ANOVA and Dunnett's *post hoc* test; AUC: *P<0.05, **P<0.01 versus DVC saline+7d STZ/Nic/HFD rats determined by ANOVA and Dunnet's *post hoc* test. Data are shown as the mean+s.e.m.

Next, we evaluated the effects of DVC ALX infusion in a rat model of type 2 diabetes (Fig. 4a) that is considered a better representation of humans with type 2 diabetes^38^. The rats were injected with nicotinamide (Nic) and low-dose streptozotocin (STZ) to prevent beta-cell compensation for HFD-induced insulin resistance, and maintained on a HFD for 7 days (Fig. 4a). We have confirmed that these 7-d STZ/Nic/HFD rats have fasting hyperglycaemia (Fig. 4b) and validated, in parallel, in our research facility to exhibit elevated hepatic glucose production^20^,^21^. In addition, diabetic 7-d STZ/Nic/HFD+DVC saline-infused rats are glucose intolerant as they have markedly elevated total glucose excursions during ivGTT compared with their non-diabetic regular chow fed DVC saline-infused counterparts (Fig. 4c). Interestingly, ALX infusion into the DVC markedly lowers total glucose excursions in diabetic rats compared with DVC saline infusion (Fig. 4c). Thus, these experiments indicate a gluco-regulatory therapeutic potential for DVC GlyT1 inhibition in high-fat-fed or diabetic rodents.

### Metabolic benefits of DVC GlyT1 inhibition in obesity

**Figure 5:**
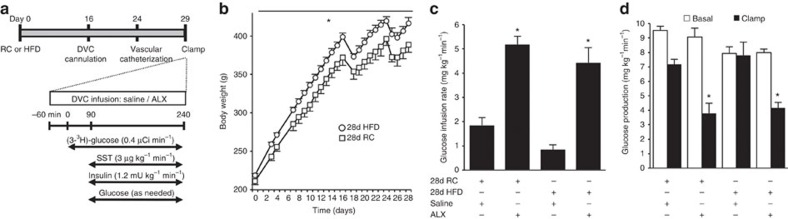
Chemical inhibition of DVC GlyT1 regulates glucose homeostasis in obese rats. (**a**) Experimental protocol for **b**–**d**. (**b**) Body weight gain in rats that were fed with HFD (white circles, *n*=18) or regular chow (RC, white squares, *n*=6). Inflections of the body weight curves at day 16 and day 24 represent DVC cannulation and vascular catheterization surgery days, respectively. *P<0.02 main effect of diet, *F*(1,22)=6.964 determined by repeated measures ANOVA. (**c**) Glucose infusion rates and (**d**) glucose production during clamps in 28d RC-fed rats with DVC infusion of saline (*n*=5) or ALX (*n*=5) and in 28-d HFD-fed rats with DVC infusion of saline (*n*=7) or ALX (*n*=7; **c**,**d**: *P<0.01 versus the respective DVC saline determined by ANOVA and Tukey's *post hoc* test). Data are shown as the mean + s.e.m.

We next assessed whether DVC GlyT1 inhibition improves glucose metabolism in 28-d HFD-induced obese rats (Fig. 5a). The rats fed a HFD for 28 days were first confirmed to be obese (Fig. 5b) and hyperinsulinemic (28d HFD rats (2.5±0.2, *n*=10) versus 28d RC rats (1.9±0.2, *n*=9), *P*<0.05, *t*-test), consistent with the fact that this obese model was validated in parallel under hyperinsulinemic–euglycaemic clamp conditions in our research facility to exhibit hepatic and peripheral insulin resistance^20^. Importantly, in both 28-day regular chow and HFD cohorts, we here report that acute inhibition of DVC GlyT1 with ALX infusion into the DVC increases glucose infusion rates (Fig. 5c) and lowers glucose production (Fig. 5d) independent of changes in the glucose uptake (Supplementary Fig. 6a) and plasma glucose levels (Supplementary Fig. 6b) during pancreatic (basal insulin)–euglycaemic clamp conditions. Notably, the glucose production-lowering effect of acute DVC GlyT1 antagonism is evident in spite of the weight gain incurred by chronic high-fat feeding (body weight on the morning of clamp experiments: 419±7 versus 390±10 g, *P*<0.05 28-d HFD versus 28-d RC, *t*-test).

**Figure 6:**
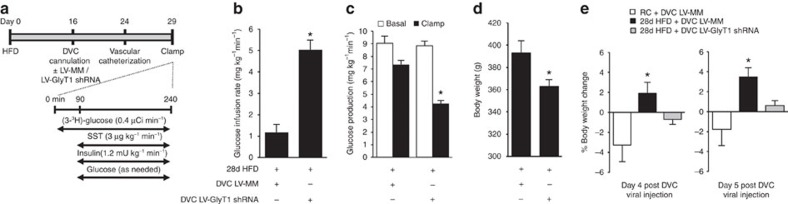
Molecular inhibition of DVC GlyT1 regulates metabolic homeostasis in obese rats. (**a**) Experimental protocol for **b**–**e**. (**b**) Glucose infusion rates and (**c**) glucose production during clamps in 28-d HFD-fed rats with DVC lentivirus (LV) injection of GlyT1 shRNA (*n*=10) or a mismatch sequence (MM, *n*=9) as a control. (**b**,**c**: *P<0.001 versus 28d-HFD+MM determined by *t*-test.) (**d**) Body weights on the morning of clamp experiments in 28-d HFD-fed rats with DVC LV MM or GlyT1 shRNA. *P<0.04 determined by *t*-test. (**e**) Percentage body weight change on days 4 and 5 following DVC injection of LV-MM (white circles, *n*=9) or GlyT1 shRNA (black circles, *n*=9) in 28-d HFD-fed rats and of MM fed with regular chow (white squares, *n*=5). *P<0.05 compared with all other groups determined by ANOVA and Dunnett's *post hoc* test. Data are shown as the mean+s.e.m.

Given that acute inhibition of DVC GlyT1 improves glucose homeostasis in short-term (Fig. 3c) and long-term high-fat-fed rats, we postulated that chronic inhibition of GlyT1 in the DVC might confer a gluco-regulatory benefit during 28 days of HFD-induced obesity. We tested this hypothesis by subjecting 28-d HFD-fed rats to targeted knockdown of GlyT1 in the DVC (via DVC LV-GlyT1 shRNA injection on day 16 after HFD; Fig. 6a) to determine whether this chronic (from day 16 to day 29; Fig. 6a) intervention modulates glucose homeostasis. Indeed, chronic genetic inhibition of GlyT1 in the DVC robustly increases the glucose infusion rate (Fig. 6b) and suppresses glucose production (Fig. 6c) as compared with MM controls. This glucose-lowering effect occurs independent of changes in glucose uptake (Supplementary Fig. 6c) and plasma glucose levels (Supplementary Fig. 6d). Surprisingly, body weights on the morning of clamp experiments are markedly lower in 28-d HFD rats with chronic DVC GlyT1 inhibition (Fig. 6d). In fact, this lowering of body weight in 28-d HFD-induced obese rats is evident by day 4 post viral (LV-GlyT1 shRNA versus LV-MM) injection (Fig. 6e). However, it is unlikely that the gluco-regulatory improvement results from a decrease in body weight since chronic DVC GlyT1 inhibition lowers glucose production in healthy rats without affecting the body weight on the morning of the clamp studies (Fig. 2f, Supplementary Fig. 4c).

### DVC GlyT1 inhibition regulates energy balance

**Figure 7:**
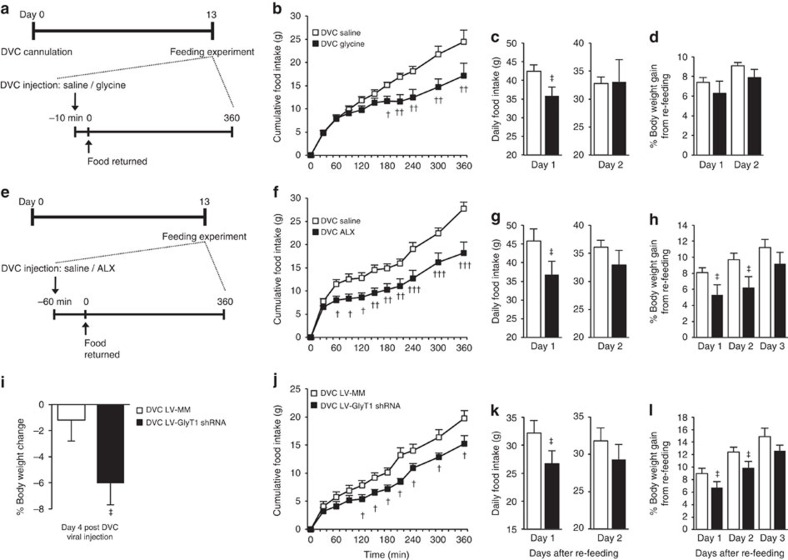
Chemical and molecular inhibition of DVC GlyT1 regulates energy balance. (**a**) Experimental protocol for feeding experiments in rats that received DVC injection of glycine (black squares, *n*=11) or saline (white squares, *n*=11). (**b**) Cumulative food intake during the feeding experiment. (**c**) Daily food intake on day 1 and day 2 after food was returned during the feeding experiment. (**d**) Percentage body weight gain on day 1 or day 2 after the feeding experiment. (**e**) Experimental protocol for feeding experiments in the rats that received DVC injection of ALX (black squares, *n*=8) or saline (white squares, *n*=8). (**f**) Cumulative food intake during the feeding experiment. (**g**) Daily food intake on day 1 and day 2 after food was returned during the feeding experiment. (**h**) Percentage body weight gain on day 1, day 2 or day 3 after the feeding experiment. (**i**) Percentage body weight change on day 4 following DVC injection of LV-MM (*n*=8) or GlyT1 shRNA (*n*=10) in regular chow-fed rats. (**j**) Cumulative food intake during the feeding experiment in rats injected with LV-MM (*n*=6) or GlyT1 shRNA (*n*=7). (**k**) Daily food intake on day 1 and day 2 after food was returned during the feeding experiment. (**l**) Percentage body weight gain on day 1, day 2 or day 3 after the feeding experiment. ^†^*P*<0.05, ^††^*P*<0.01, ^†††^*P*<0.001 determined by *t-*test at each time point, ^‡^*P*<0.05 determined by *t*-test at each time. Data are shown as the mean+s.e.m.

Nonetheless, it was important to next investigate whether the local elevation of glycine in the DVC associated with DVC GlyT1 inhibition can also regulate energy balance. First, we tested the direct effect of DVC glycine sensing on appetite and body weight regulation in healthy rats that received DVC surgery 13 days prior (Fig. 7a). Following a 22 h fast, injection of glycine into the DVC begins to lower food intake compared with saline-infused controls by ∼120 min post injection and refeeding (Fig. 7a,b), an effect that becomes significant by 180 min and persisted for 1 day after refeeding (Fig. 7c). DVC glycine has no significant effect on days 1 or 2 post refeeding percentage body weight gain (Fig. 7d). Second, we assessed whether chemical inhibition of GlyT1 in the DVC could recapitulate these glycine-induced satiation effects. Indeed, DVC ALX injection reduces food intake by 60 min after refeeding (or 120 min post-ALX injection; Fig. 7e,f) with the effect still present 1 day post refeeding (Fig. 7g). DVC ALX also reduces the percentage body weight gain after 1 and 2 days but not after 3 days of refeeding (Fig. 7h). Finally, we evaluated whether molecular inhibition of GlyT1 in the DVC could regulate energy balance. LV-GlyT1 shRNA or LV-MM was injected into the DVC of healthy rats to knock down DVC GlyT1, resulting in reduced body weight of LV-GlyT1 shRNA rats 4 days post viral injection compared with LV-MM (Fig. 7i), similar to the effect observed in obese rats (Fig. 6e). The viral-injected regular chow-fed rats were then subjected to a 22 h fast in the evening of day 4, and genetic knockdown of DVC GlyT1 lowers cumulative food intake as early as 120 min following refeeding (Fig. 7j) and up to 1 day post refeeding (Fig. 7k). In parallel, LV-GlyT1 shRNA versus LV-MM injection lowers the percentage body weight gain following 1 and 2 days but not 3 days post refeeding (Fig. 7l).

Taken together, we provide evidence that DVC GlyT1 inhibition and subsequent glycine elevation triggers a sensing mechanism in the DVC to lower feeding and body weight in rats.

## Discussion

We have shown that targeted inhibition of DVC GlyT1 through either administration of a GlyT1 inhibitor or a chronic molecular knockdown improves glucose homeostasis and lowers body weight gain in diabetic and obese rodents.

The effect of DVC GlyT1 inhibition on glucose production regulation requires a hepatic vagal-dependent communication between the brain and the liver. Although the neurocircuitry involved in food intake and body weight regulation by DVC GlytT1 inhibition (or glycine sensing) remains unclear, the underlying neuronal relay is likely different than glucose production regulation (since glucose production is altered by DVC GlyT1 inhibition independent of changes in food intake and body weight (Figs 1e, 2f and 5d), as well as blood pressure and heart rate regulation (since DVC injection of glycine or glutamate induce changes in blood pressure and heart rate at a much faster rate^39^,^40^ than changes in feeding induced by DVC glycine injection (Fig. 7b)). It would be important to follow up on the potential long-term control of food intake and body weight regulation via repeated injections of glycine or ALX, particularly knowing that a knockdown of DVC GlyT1 for 13 days exerts an anti-obesity effect.

Although the individual cells in the DVC involved in the metabolic control of DVC GlyT1 inhibition remain to be identified, the potentiation and activation of the GluN1/GluN2-containing NMDA receptor in the DVC is necessary for the gluco-regulatory effect of DVC GlyT1 inhibition (Figs 1, 2, 3). Given that the NMDA receptors are expressed in the plasma membrane and are necessary for the metabolic effect of DVC GlyT1 inhibition, DVC ALX infusion increases extracellular glycine levels within the DVC as assessed by microdialysis (Fig. 1h), and that DVC glycine infusion (like GlyT1 inhibition) potentiates DVC NMDA receptors to lower glucose production in healthy^23^ and 3-d HFD (Fig. 3c) rats, glycine is proposed to be the endogenous agonist that mediate the metabolic control of DVC GlyT1 inhibition. D-serine, like glycine, is also a co-agonist of the NMDA receptors. However, it is unlikely that D-serine is the endogenous agonist that mediates the effects of GlyT1 inhibition as injection of GlyT1 inhibitor elevates extracellular glycine but not serine and glutamate levels in the brain of rats^41^. Future studies are necessary to dissect the specific role of glycine versus serine *per se* as well as in the presence of GlyT1 inhibition in regulating glucose and energy homeostasis.

Although ketamine (a partial NMDA receptor antagonist) was used to anaesthetize the animals for brain and vascular surgeries, any potential confounding effects of ketamine on the gluco-regulatory studies should be absent by the time we carry out the infusion experiments as body weight and food intake of the rodents have fully recovered. In addition, MK-801 inhibits the GluN1/GluN2 but not the GluN1/GluN3 NMDA receptors^42^. Given that in our current study, DVC MK-801 fully reverses the ability of both DVC ALX infusion and DVC LV-GlyT1 shRNA viral injection to inhibit glucose production (Figs 1 and 2), it is likely that activation of the GluN1/GluN2 receptors, and not GluN1/GluN3, is essential for the metabolic effects of DVC GlyT1 inhibition and glycine sensing. Consistent with this hypothesis, bi-directional changes of NMDA receptors in the DVC via DVC infusion of NMDA or NMDA receptor antagonist AP5 alter glucose production^23^, while strychnine-sensitive glycine receptors do not appear to mediate DVC glycine sensing to regulate glucose production^23^, altogether strengthening the claim that GluN1/GluN2-containing NMDA receptors mediate the glucose-lowering effect of DVC GlyT1 inhibition. Nonetheless, a role for DVC GluN1/GluN3 NMDA receptor in glucose regulation remains to be directly assessed.

DVC GlyT1 inhibition improves glucose tolerance independent of a rise in plasma insulin levels and lowers glucose production when insulin levels are maintained at basal during the pancreatic clamps. Thus, it is tempting to speculate that DVC GlyT1 inhibition may improve glucose homeostasis in type 1 diabetic insulin-deficient conditions, particularly knowing that leptin action in the brain and the gut, as well as nutrient sensing in the gut, have been documented to improve glucose homeostasis in insulin-deficient type 1 diabetic rodents^19^,^43^,^44^,^45^. This working hypothesis warrants future investigation. On the other hand, it would also be of future interest to assess whether DVC GlyT1 inhibition reverses insulin resistance in type 2 diabetic and obese rodents using the hyperinsulinemic–euglycaemic clamp technique to achieve insulin-stimulated conditions.

The finding that systemic administration of ALX can recapitulate the glucose production-lowering effect of GlyT1 inhibition in the DVC during the pancreatic (basal insulin)–euglycaemic clamp setting further substantiates the potential therapeutic relevance of GlyT1 inhibitors in diabetes and obesity. However, given that NMDA receptors are also expressed in the islets and alter glucose-stimulated insulin secretion^46^, future studies are warranted to investigate the short- and long-term metabolic benefits of ALX administration in non-clamp conditions.

Our current set of findings serve as proof of concept for the potential of GlyT1 inhibition as a singular therapeutic target for the concurrent treatment of both diabetes and obesity, in addition to its current use in the treatment of schizophrenia. Interestingly, patients with schizophrenia have over four times the risk for abdominal obesity and twice the risk for diabetes compared with general population controls^47^, highlighting the possibility that common pathologies may contribute to the development of these diseases.

Among several GlyT1 inhibitors that have undergone clinical trials, bitopertin has seen the most success by advancing to phase III trials for the treatment of schizophrenia^26^,^27^. ALX, on the other hand, has demonstrated relatively poorer tolerance *in vivo*^48^. However, although ALX never entered clinical trials, it is extensively used as a pharmacological tool for the study of glycine transporter function^48^. Interestingly, DVC administration of ALX in the present study increases DVC extracellular glycine levels, which mimics a comparable effect of a low oral dose of bitopertin on CSF glycine levels in rats^49^. These two drugs may therefore trigger a similar degree of NMDA receptor-mediated neurotransmissions to elicit comparable metabolic effects. Given that several GlyT1 inhibitors have successfully demonstrated safety and efficacy in humans and that systemic ALX infusion recapitulates the ability of ALX infusion into the DVC to lower glucose production in HFD rats, we propose that GlyT1 inhibitors be considered as pharmacological agents for the restoration of glucose and energy homeostasis in obesity and diabetes.

## Methods

### Animal preparation and surgical procedures

Male Sprague Dawley rats (Charles River Laboratories, Saint-Constant, QC, Canada) weighing 280–300 g (9-week old) were used. For chronic 28-day feeding studies (see below), a separate set of rats initially weighing 200–220 g and fed with regular chow or a high-fat diet were used. The rats were individually housed, subjected to a standard light–dark (7:00 light, 19:00 dark) cycle, and had *ad libitum* access to drinking water and standard regular chow or a 10% lard-enriched chow (high-fat diet, HFD) where indicated (see below). The rats were anaesthetized during surgeries (ketamine, 60 mg kg^−1^; xylazine, 8 mg kg^−1^). Bilateral, 26-gauge, stainless steel guide cannulae (Plastics One Inc, Roanoke, VA, USA) were stereotaxically implanted into the DVC via coordinates targeting the nucleus of the solitary tract within the DVC (NTS, 0 mm on the occipital crest, 0.4 mm lateral to the midline, 7.9 mm below the cranial surface; Supplementary Fig. 3)^50^. Eight days following DVC surgery, indwelling catheters were surgically implanted in the left carotid artery and right jugular vein for blood sampling and infusions, respectively^51^. Post-surgical body weight and food intake were monitored daily. The rats attained a minimum of 90% of their pre-vascular surgery body weight before undergoing experimentation 5 days following vascular surgery. The rats that did not fully recover were excluded from the study. The rats were randomly designated into groups before experiment and no blinding was done.

In parallel, microdialysis studies were performed on male Sprague Dawley rats (Charles River, Raleigh, NC, USA), which started with a body weight of ∼280–300 g and were individually housed in the Yale Animal Resources Center in temperature (22–23 °C) and humidity-controlled rooms. The animals had free access to rat chow (Harlan Teklad, Indianapolis, IN, USA) and water. On arrival at the Yale Animal Resources Center, the animals were acclimatized to handling and a 12-h light cycle (lights on between 7:00 h and 19:00 h) for 1 week before experimental manipulation. The principles of laboratory animal care were followed, and the experimental protocols were approved by the Institutional Animal Care & Use Committee at the Yale University. The rats were anaesthetized with isoflurane and the heads were positioned into a stereotaxic frame (David Kopf Instruments, Tujunga, CA, USA). A single stainless steel guide cannula for microinjection and microdialysis (Eicom Corporation, Japan) was implanted intracranially using the following stereotaxic co-ordinates from Paxinos and Watson (0 mm on the occipital crest, 5 mm medial–lateral and 7.4 mm ventral at an angle of 35° for microdialysis). This targeted the 1 mm microdialysis probe (Eicom Corporation, Japan) to the NTS within the DVC.

The male C57BL/6 mice (Jackson Laboratory, Bar Harbor, ME, USA) at 18 weeks of age were housed in a standard light–dark cycle with *ad libitium* access to drinking water and standard chow. The mice were anaesthetized during stereotaxic and vascular surgeries (Avertin, 0.6 mg g^−1^). A unilateral, 33-gauge, stainless steel guide cannula (Plastics One Inc) was stereotaxically implanted into the fourth ventricle (ICV-4; 6.0 mm posterior to Bregma, 4.0 mm below the cranial surface)^52^,^53^. One week following ICV-4 surgery, an indwelling catheter was surgically implanted in the right jugular vein^52^,^53^. Post-surgical body weight and food intake were monitored daily. The mice attained a minimum of 90% of their pre-vascular surgery body weight before undergoing pancreatic clamp 3–5 days following vascular surgery.

All the experimental animal procedures in this study were reviewed and approved by the Institutional Animal Care and Use Committee of the UHN.

### Intravenous glucose tolerance test

The experiments were performed in overnight-fasted (16 to 18 h) male Sprague Dawley rats 5 days after vascular catheterization. The basal blood samples were obtained in conscious, unrestrained rats immediately before the start of DVC infusions (0.33 μl h^−1^, CMA 400 syringe pump, CMA Microdialysis, Inc., North Chelmsford, MA, USA) of 0.9% saline or ALX (ALX 5407, Tocris Bioscience, 40 nM), which were commenced at *t*=−240 min and maintained until the end of the experiment at *t*=60 min to ensure that rats received the same duration of DVC treatment as clamp experiments (see below). After *t*=0 min, the blood samples were obtained, an intravenous bolus of glucose (20% glucose, 0.25 g kg^−1^) was injected and flushed with saline. The injections were administered via the jugular vein catheter, and the blood was sampled from the carotid artery catheter to measure plasma glucose and insulin levels for 60 min following glucose injection as described^53^.

### Pancreatic basal insulin euglycaemic clamp in rats

The experiments were performed in male Sprague Dawley rats fasted for ∼4–6 h before clamp experiments to ensure comparable post-absorptive nutritional status. The basal blood samples were obtained in conscious, unrestrained rats immediately before the start of DVC infusions (0.33 μl h^−1^) of the following infusates: (i) 0.9% saline, (ii) MK801 (0.06 ng min^−1^, dissolved in saline), (iii) 7CKNA (7-chlorokynurenic acid, 30 μM, dissolved in saline), (iii) ALX (ALX 5407, Tocris Bioscience, 40 nM, dissolved in saline), (iv) ALX (40 nM)+MK801 (0.06 ng min^−1^), (v) ALX (40 nM)+7CKNA (30 μM), (vi) glycine (10 μM, dissolved in saline). Using the same DVC infusion protocol as the current study, glycine at 10 μM was validated to elevate DVC glycine levels by ∼1.2-fold and lower glucose production^23^ and secretion of triglyceride-rich very-low-density lipoproteins (VLDL-TG)^50^ in regular chow-fed healthy rats, while MK-801 at 0.06 ng min^−1^ and 30 μM of 7CKNA blocked the effects of DVC glycine infusion to lower glucose production^23^ and VLDL-TG secretion^50^. Thus, we have chosen to use these same dose and concentrations for the inhibitors in this study examining the effect of DVC GlyT1 inhibition (likely mediating glycine sensing). In fact, a total amount of ∼20 ng of MK-801 was delivered into the DVC over the course of 330 min in the current studies, which is comparable to the 50 ng of MK-801 delivered into the NTS (or DVC) that regulated feeding behaviour^54^. Similarly, our concentration of 30 μM 7CKNA was also well within the range reported by the other studies that have indicated that 10–50 μM of 7CKNA reduces 80–90% of glycine binding to rat cerebral cortex synaptic plasma membrane^55^, while 30 μM of 7CKNA inhibits NMDA-induced transmitter release from rat hippocampal slices^56^. More importantly, DVC infusion of neither MK-801 at 0.06 ng min^−1^ nor 30 μM 7CKNA *per se* resulted in increased glucose production but only blocked the effect of DVC glycine infusion to lower glucose production^23^. Thus, any concern for the non-specific effects of these inhibitors in regulating glucose homeostasis can be safely excluded. The concentration of 40 nM ALX was chosen based on the IC50 of ALX for GlyT1 (4 nM; ref. 29) and factoring into a dilution factor when chemical inhibitors are infused into the DVC. Infusions of MK801 or 7CKNA, when used, or saline as a control, were commenced at *t*=−90 min; infusions of ALX±MK801 or 7CKNA were commenced at *t*=−60 min and infusions of glycine±7CKNA were commenced at *t*=0 min and maintained for the duration of the experiment. ALX, an inhibitor of the GlyT1 transporter, inhibits the binding of glycine to its cellular transporter and elevates extracellular levels of glycine^41^. ALX infusion was initiated earlier to allow for extracellular levels of glycine in the DVC to accumulate. Clamp methodology was performed as follows^53^. A primed, continuous infusion (PHD2000 syringe pump, Harvard Apparatus, Saint Laurent, QC, Canada) of [3-^3^H]-glucose (PerkinElmer; 40 μCi bolus+0.4 μCi infusion) was commenced at *t*=0 min and maintained until the end of the clamp experiment at *t*=240 min to measure glucose kinetics using tracer-dilution methodology. The glucose turnover was calculated using steady-state formulae, in which the rate of appearance of glucose is calculated using [3-^3^H]-glucose. The total rate of appearance of endogenous glucose production is equivalent to the rate of glucose utilization during the basal period (*t*=60–90 min). The pancreatic basal insulin–euglycaemic clamp was initiated at *t*=90 min with the primed continuous infusion of insulin (1.2 mU kg^−1^ min^−1^, somatostatin (SST, 3 μg kg^−1^ min^−1^) and a variable infusion of 25% glucose to maintain glycemia at a similar level to the basal period and was maintained until *t*=240 min. Plasma samples were obtained every 10 min for the determination of [3-^3^H]-glucose specific activity and glucose levels. Wedges containing the DVC, the left and right portions of spinal trigeminal tr. (sp5), Spinal 5nu caudal part (Sp5C), Spinal 5nu, interpolar (Sp5I) and the pyramidal tr. (py) (Supplementary Fig. 3i–v, see ‘Brain tissue sampling' section below) were collected immediately after the experiments, frozen in liquid nitrogen and stored at −80 °C for analysis.

### Hepatic branch vagotomy in rats

A separate set of male Sprague Dawley rats underwent hepatic branch vagotomy^23^ on the same day as vascular catheterization surgeries. The hepatic branch of the ventral subdiaphragmatic vagal trunk was transected, and the omentum between the liver and the esophagus was severed such that any tissue connections between the liver and the esophagus were removed. The neural communication between the central nervous system and the liver was disrupted upon transection of the hepatic vagal nerve, and to a much lesser degree, the innervations to the gut were also disrupted. The sham-operated rats underwent similar procedure except for transection of the vagus. After surgical recovery, the rats underwent clamp experiments as described above.

### Microdialysis

One week after surgery, the male Sprague Dawley rats were fasted for 4–6 h before the experiments to ensure comparable post-absorptive nutritional status. On the day of the study, the microdialysis-microinjection probe was inserted through the guide cannula and the animals were allowed to recover for 2.5 h before collection of the baseline sample (Supplementary Fig. 1b). Artificial extracellular fluid was perfused through the probe at a rate of 0.5 μl min^−1^ throughout the study. Following the recovery period, we collected a baseline sample over the course of 2 h before the start of ALX (40 nM in saline, Tocris Bioscience) infusion. The ALX was infused into the DVC (*via* the microinjection needle) at a rate of 0.33 μl h^−1^ for a total duration of 300 min. The microdialysate samples were collected at 60, 180 and 300 min following the start of ALX infusion. The control animals were infused with saline and sampled under similar conditions. At the end of the study, the animals were killed with an overdose of sodium pentobarbital.

### DVC virus injection

Immediately after stereotaxic surgery while anaesthetized, 3 μl of adenovirus or lentivirus were injected over 30 s in each of the DVC cannulae with microsyringes. An adenovirus expressing shRNA to the GluN1 subunit of the NMDA receptor (Ad- GluN1 shRNA, 4.0 × 10^11^ p.f.u.  ml^−1^) or a mismatch sequence as a control (Ad-MM, 4.0 × 10^11^ p.f.u.  ml^−1^) was injected into the DVC for one set of experiments using the same protocol that we have validated^23^. This adenoviral GluN1 shRNA knockdown procedure decreases GluN1 protein levels specifically in the DVC region^23^. In separate sets of experiments, a lentivirus expressing shRNA to GlyT1 (LV-GlyT1 shRNA, 1.0 × 10^6^ infectious units; sc-270432-V, Santa Cruz Biotechnology, Inc., Dallas, TX, USA) or a mismatch sequence as a control (LV-MM, 1.0 × 10^6^ infectious units; sc-108080, Santa Cruz Biotechnology) was injected in the DVC. Eight days after DVC cannulation and virus injection, vascular catheterization was performed as described above in rats that would undergo clamp experiments. Thirteen days after DVC cannulation, virus-injected rats underwent clamp or feeding experiments as described above.

### Brain tissue sampling in rats

At the end of the experiments, the rats were injected with 3 μl bromophenol blue through each side of the bilateral DVC catheter to verify the correct placement of the catheter for the viral injection studies, while bromophenol blue was co-infused with chemical reagents for the non-viral injection studies. Once the whole brain is harvested from the anaesthetized rat via decapitation, the cerbellum is lifted to expose the caudal part of the brain (Supplementary Fig. 3i–v). Only those data for rats that showed injection of dye within the vagal triangle (Supplementary Fig. 3ii–v) were included. A spatula was used to extract the section of vagal triangle overlaying the DVC (blue; Supplementary Fig. 3ii–v). In addition, sections of tissues were dissected out from the left (yellow) and right (purple) lateral regions of the caudal brain containing sp5 (spinal trigeminal tr.), Sp5C (spinal 5nu, caudal part), Sp5I (spinal 5nu, interpolar), as well from the lower region containing py (pyramidal tr) of the distal caudal brain (green; Supplementary Fig. 3ii–v).

### Microdialysate sample glycine analysis

Microdialysate samples collected at baseline, 180 and 300 min were analysed using a fluorometric assay kit (Glycine assay kit (Fluorometric), #K589-100, Biovision Incorporated, Milpitas, CA, USA) according to the manufacturer's directions. A sample volume of 50 μl was used in the assay and since glycine content was low, each microdialysate sample was spiked with 0.3 nmol of the glycine standard to bring the values within a more reliable reading range of the assay and the calculations were adjusted accordingly. Since glycine levels were relatively stable during ALX infusion, the concentrations obtained from the last two microdialysate samples were averaged together and compared with the baseline levels. Similar calculations were performed for the control animals.

### Acute (3-day) and chronic (28-day) high-fat feeding in rats

Two separate sets of male Sprague Dawley rats were fed a palatable, 10% lard-enriched HFD (TestDiet #571 R, Purina Mills, Richmond, IN, USA), either for 3 days (acute, 3-d HFD) or 28 days (chronic, 28-d HFD) before the clamp experiments. The composition of the HFD (3.9 kcal g^−1^) differs from regular chow (3.1 kcal g^−1^): fat content (34% versus 18%); protein (22% versus 33%) and carbohydrate (44% versus 49%) content. A 28-day regular chow-fed cohort of rats was used in parallel to the 28-d HFD group. Both cohorts of HFD rats underwent the pancreatic clamp experiments as described above. The rats that did not overeat were excluded from the studies.

### Intravenous ALX infusion clamps

In a separate group of 3-d HFD-fed male Sprague Dawley rats, the pancreatic clamp experiments were performed as described above, with the exception that a continuous intravenous ALX (4.1 μg kg^−1^ min^−1^, dissolved in 6% DMSO infused at 20 μl min^−1^) or intravenous 6% DMSO (20 μl min^−1^) as vehicle was initiated at *t*=−90 min. At *t*=0 min, a primed continuous infusion of 3[H^3^]- glucose was commenced and maintained until the end of the experiment, *t*=210 min. At *t*=90 min, the pancreatic basal insulin clamp was initiated with the primed continuous infusion of insulin (1.2 mU kg^−1^ min^−1^), somatostatin (SST, 3 μg kg^−1^ min^−1^), and a variable infusion of 25% glucose to achieve euglycaemia was administered until *t*=210 min. Intravenous ALX was constantly infused at 4.1 μg kg^−1^ min^−1^ to achieve a total amount of 1.23 mg kg^−1^ ALX delivered into the blood in 300 min. This choice of dose is based on the fact that intravenous ALX injected at 1-2 mg kg^−1^ has been documented to potentiate NMDA-evoked firing in PFC neurons of rats *in vivo*^57^.

### Induction of experimental type 2 diabetes

Six days after DVC surgery, a separate set of male Sprague Dawley rats was given an intraperitoneal injection of nicotinamide (Nic, 170 mg kg^−1^) followed by an intraperitoneal low-dose injection of streptozotocin (STZ, 65 mg kg^−1^) 15 min later and fed with a HFD for 7 days as described^20^,^21^,^38^, before intravenous glucose tolerance tests as described above. The rats that did not present with fed hyperglycaemia (for example, >9 mM) were excluded from the study.

### Fasting–refeeding experiments

Separate groups of male Sprague Dawley rats were subjected to a 22-h fast (food removed at 7 pm) before undergoing the refeeding experiment. DVC injections (0.04 μl min^−1^ for 5 min using CMA syringe pumps) of 0.9% saline, ALX (40 nM) or glycine (10 μM) were given at *t*=−60 min (ALX) or *t*=−10 min (glycine; Fig. 7a,e). To prevent backflow of the injected volume, injection cannulae were left in guide cannulae for an additional 5 min with the pump off, and dummy cannulae are re-inserted and secured with dust caps. Regular chow was returned to cages at 17:00 h, *t*=0 min. The food intake was measured every 30 min for the first 4 h of the refeeding experiment, and every 1 h until *t*=360 min. Food intake and body weight were measured again 20 h (day 1) and 44 h (day 2) after the rats were refed.

### Pancreatic basal insulin euglycaemic clamp in mice

The experiments were performed in male C57BL/6 mice fasted for ∼4–6 h before clamp experiments to ensure comparable post-absorptive nutritional status. The basal blood samples were obtained in conscious, unrestrained mice immediately before the start of ICV-4 infusions (1.02 μl h^−1^) of saline or ALX (40 nM), which were commenced at *t*=−90 min and maintained for the duration of the experiment. The clamp methodology in mice was performed as follows^53^. A primed, continuous infusion of [3-^3^H]-glucose (1 μCi bolus+0.1 μCi infusion) was commenced at *t*=0 min and maintained until the end of the clamp experiment at *t*=180 min to measure glucose kinetics. The basal period was defined as *t*=50–60 min. The pancreatic basal insulin-euglycaemic clamp was initiated at *t*=60 min with a primed continuous infusion of insulin (1.4 mU kg^−1^ min^−1^, SST (8.3 μg kg^−1^ min^−1^), and a variable infusion of 10% glucose to maintain glycaemia at a similar level to the basal period and was maintained until *t*=180 min. The plasma samples were obtained every 10 min for the determination of [3-^3^H]-glucose specific activity and glucose levels.

### Western blot analyses

GlyT1 protein levels were measured in purified plasma membrane fractions of brain tissue wedges from the rats that received LV GlyT1 shRNA or LV MM injections. The brain tissue wedges were collected 13 days following lentivirus injection and immediately frozen in liquid nitrogen and stored at −80 °C until analysis. Purified plasma membrane protein fractions were isolated using a commercial kit suitable for mammalian tissues (Plasma Membrane Protein Extraction Kit #K268-50, BioVision Incorporated, Milpitas, CA, USA)^51^. Purified plasma membrane fraction protein concentrations were measured using a BCA Protein Assay kit (#K812-1000, BioVision Incorporated) and 6 μg of protein was subjected to electrophoresis on 8% polyacrylamide gels and transferred to nitrocellulose membranes. The membranes were incubated with blocking solution (5% BSA in Tris-buffered saline containing 0.2% Tween-20 (TBS-T)) for 1 h at room temperature and overnight at 4 °C in primary antibody solutions diluted 1/1,000 in 5% BSA in TBS-T of GlyT1 (ab113823 rabbit, Abcam, Cambridge, MA, USA), or insulin receptor (IR) β (L55B10 mouse, #3020, Cell Signaling Technology, Danvers, MA, USA) after 10 min shaking in antibody stripping buffer (Gene Bio-Application Ltd, Yavne, Israel) and re-blocked as above. Protein expression was detected using an HRP-linked secondary antibody (rabbit and mouse, respectively, diluted 1/4,000 in blocking solution) and an enhanced chemoluminescence reagent (Pierce ECL Western Blotting Substrate, Thermo Scientific, Rockford, IL, USA). Immunoblots were detected using a MicroChemi 4.2 chemiluminescent imaging system and quantified with GelQuant image analysis software (DNR Bio-Imaging Systems, Jerusalem, Israel). Plasma membrane GlyT1 protein levels were normalized to the plasma membrane protein levels of IR. See Supplementary Fig. 7 for uncropped blots.

### Biochemical analysis

Plasma glucose concentrations were measured by the glucose oxidase method (Glucose Analyzer GM9, Analox Instruments, Lunenburg, MA, USA). Plasma insulin levels were determined by radioimmunoassay (Millipore Canada Ltd, Etobicoke, ON, Canada).

### Calculations and statistics

The sample size for each group was chosen on the basis of study feasibility and prior knowledge of statistical power form previously published experiments. For pancreatic clamp experiments in rats, measurements during *t*=60–90 min were averaged for the basal period, and *t*=210–240 min, and where possible *t*=180–210 min were averaged for the clamp period. In mice, measurements during *t*=50–60 min were averaged for the basal period and *t*=160–180 min were averaged for the clamp period. Integration of the area under the curve was calculated with GraphPad Prism 6 software (LaJolla, CA, USA). Unpaired Student's *t*-tests were performed in the statistical analysis of two groups. Where comparisons were made across more than two groups, analysis of variance (ANOVA) was performed, and if significant, was followed by Dunnett's or Tukey's *post hoc* tests when appropriate. The measurements that were taken repeatedly over time were compared using repeated-measures ANOVA; if the time and treatment interaction between groups was found to be significant, Sidak's multiple comparisons test or *t*-tests were used to determine the statistical significance at specific time points between groups. Differences in the overall effects of HFD diet on body weight are indicated where significance was found following repeated-measures ANOVA. The *P* value <0.05 was considered statistically significant.

### Data availability

All the relevant data are available from the authors on request and/or are included within the manuscript (and its Supplementary Information files).

## Additional information

**How to cite this article**: Yue, J. T. Y. *et al*. Inhibition of glycine transporter-1 in the dorsal vagal complex improves metabolic homeostasis in diabetes and obesity. *Nat. Commun.* **7**, 13501 doi: 10.1038/ncomms13501 (2016).

**Publisher's note**: Springer Nature remains neutral with regard to jurisdictional claims in published maps and institutional affiliations.

## Supplementary Material

Supplementary Information Supplementary Figures 1-7.
