# Supplementary material for: Inhibition of glycine transporter-1 in the dorsal vagal complex improves metabolic homeostasis in diabetes and obesity
Source: Nat Commun. 2016 Nov 22;7:13501. doi: 10.1038/ncomms13501 (PMC5121412; doi:10.1038/ncomms13501)
Supplement: Supplementary Information — Supplementary Figures 1-7. [file ncomms13501-s1.pdf]

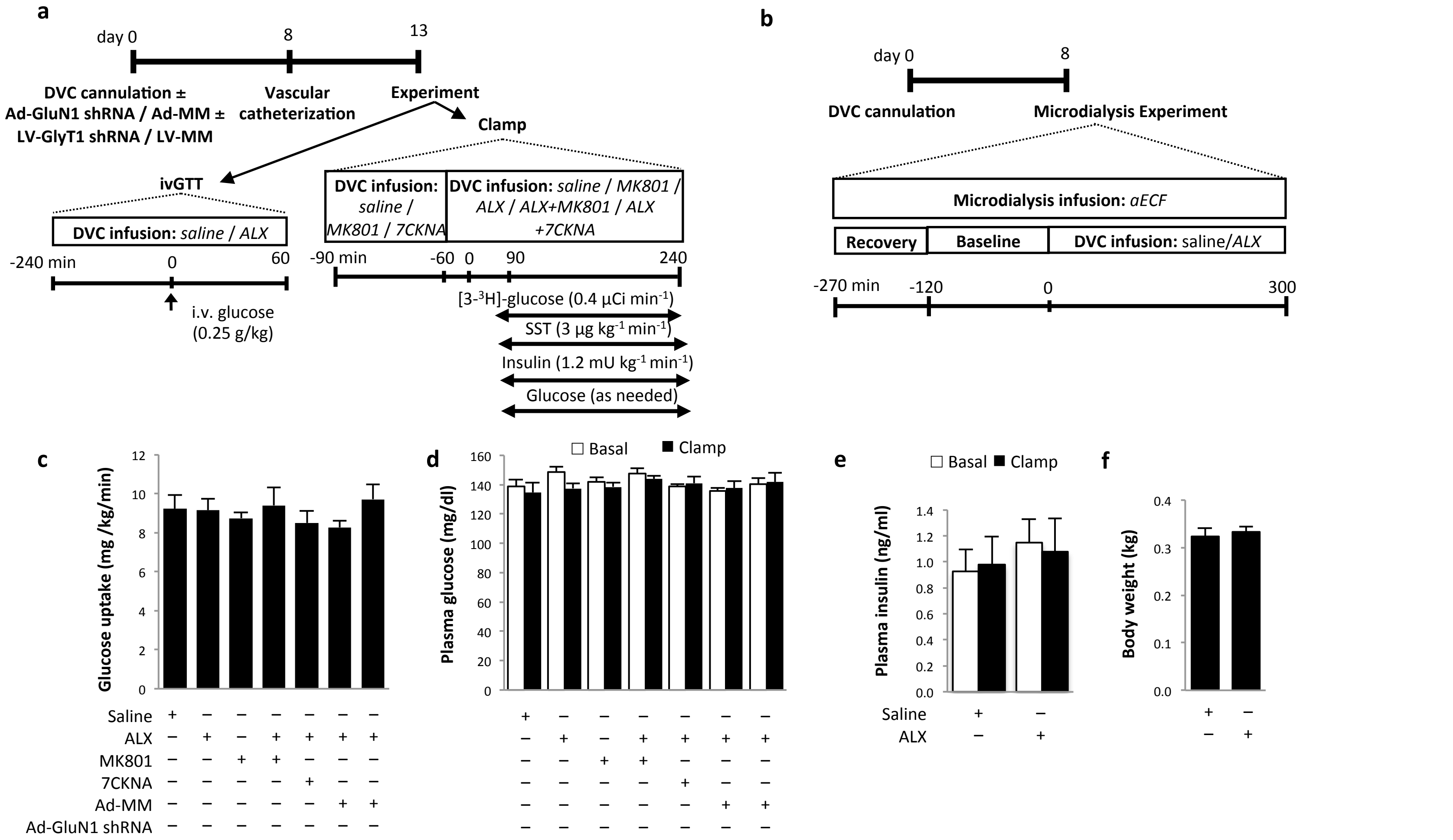

Supplementary Figure 1. Metabolic effects of chemical inhibition of DVC GlyT1 in healthy rats.
(a) Experimental protocols for experiments shown in Fig. 1. In intravenous glucose tolerance tests (ivGTT), infusions of saline or ALX into the DVC commenced at t=-240min and were maintained for the duration of the experiment. In clamps experiments, pre-infusions of saline, MK801, or 7CKNA into the DVC commenced at t=-90min. Infusions of saline, MK801, ALX, ALX+MK801, or ALX+7CKNA into the DVC commenced at t=-60min and were maintained for the duration of the clamps. **(b)** Experimental protocol for microdialysis studies shown in Fig. 1i. **(c)** Glucose uptake, **(d)** basal and clamp plasma glucose levels during clamps with DVC infusion of saline (n=11), ALX (n=9), MK801 (n=9), ALX+MK801 (n=5), ALX+7CKNA (n=5), Ad-MM+ALX (n=5), or Ad-GluN1 shRNA+ALX (n=5), **(e)** basal and clamp plasma insulin levels during DVC infusion of saline (n=6) or ALX (n=6), and **(f)** body weights on the morning of clamp experiments before DVC infusion of saline (n=11) or ALX (n=9). Data are shown as the mean + SEM.

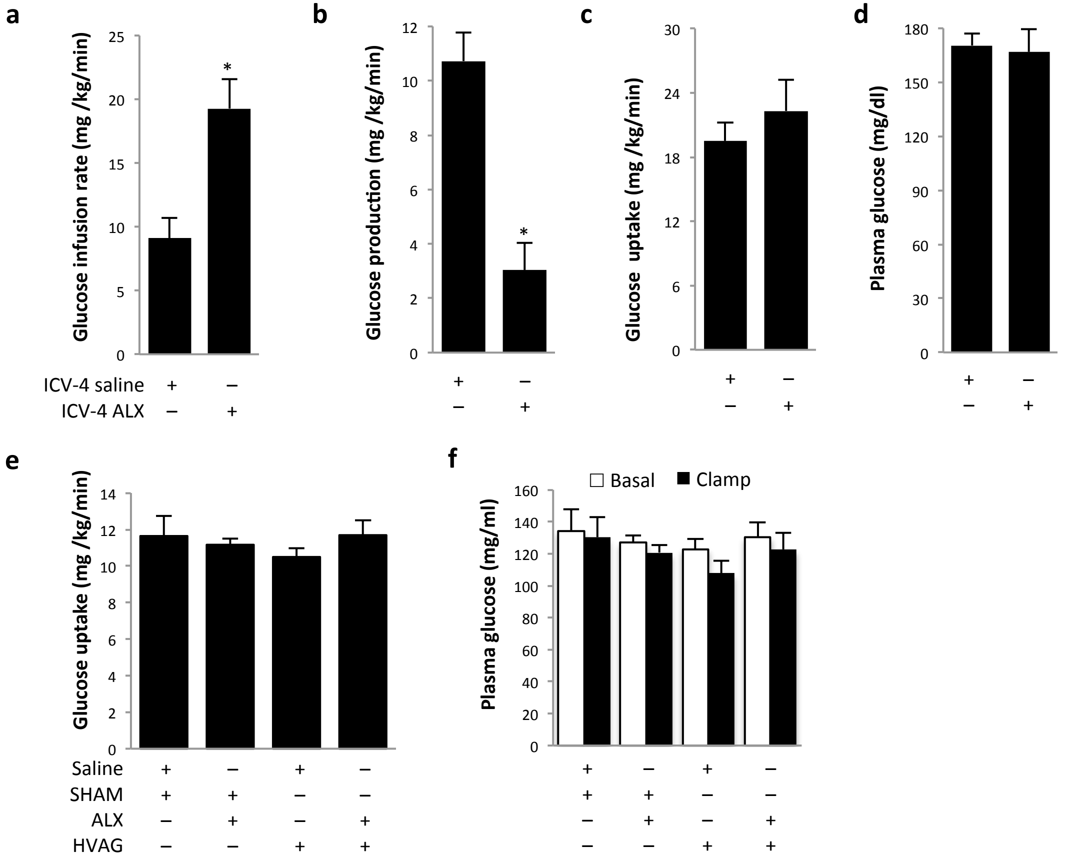

Supplementary Figure 2 . Metabolic effects of chemical inhibition of GlyT1 in the 4th ventricle of mice and in the DVC of hepatic vagotomized rats.
(a) Glucose infusion rates, **(b)** rates of clamp glucose production, **(c)** glucose uptake, and **(d)** clamp plasma glucose levels during clamp experiments with ICV-4th ventricle infusion of ALX (n=5) or saline (n=6) in C57BL/6 mice; *P<0.02 *vs* saline determined by *t*-test. **(e)** Glucose uptake, and **(f)** basal and clamp plasma glucose levels during clamp experiments with DVC infusion of ALX in vagotomized (n=7) or sham-operated (n=5) rats or DVC infusion of saline in vagotomized (n=7) or sham-operated rats (n=5) rats. Data are shown as the mean + SEM.

Supplementary Fig. 3

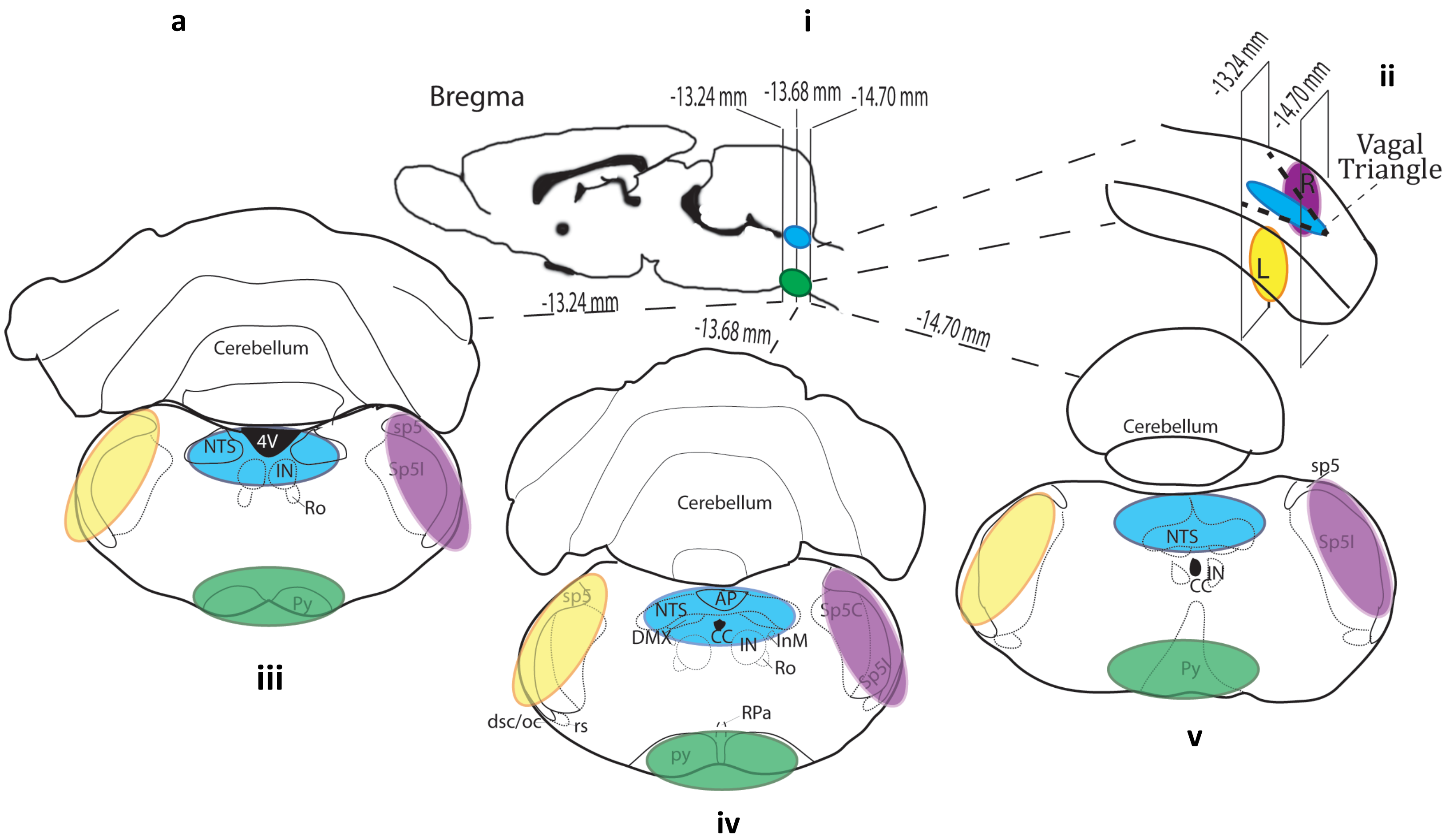

Supplementary Figure 3. Brain regions included in the GlyT1 protein analysis.
This include the DVC region (blue), the left (L) lateral region (yellow) containing spinal trigeminal tr. (sp5), spinal 5nu, caudal part (Sp5C), spinal 5nu, interpolar (Sp5I), the right (R) lateral region (purple) containing sp5, Sp5C, Sp5I and the bottom region (green) containing pyramidal tr. (py). i: Saggital image representing the rat brain. ii: Vagal triangle overlaying the DVC located in the caudal part of the brain. iii-v: three coronal images representing the proximal, medial and distal regions of the caudal brain indicating all the regions included in the analysis of the GlyT1 protein.

Supplementary Fig. 4

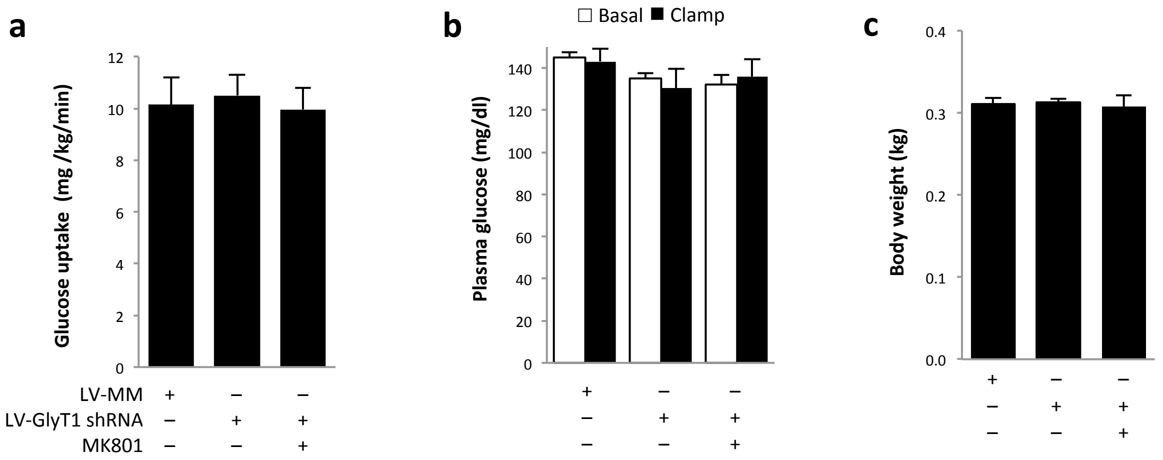

Supplementary Figure 4. Metabolic effects of molecular inhibition of DVC GlyT1 in healthy rats.
(**a**) Glucose uptake, (**b**) basal and clamp plasma glucose levels during clamps, and (**c**) body weights on the morning of clamp experiments in rats injected with DVC LV-MM (n=7), LV-GlyT1 shRNA (n=7), or LV-GlyT1 shRNA with DVC MK801 infusion (n=6). Data are shown as the mean + SEM.

Supplementary Fig. 5

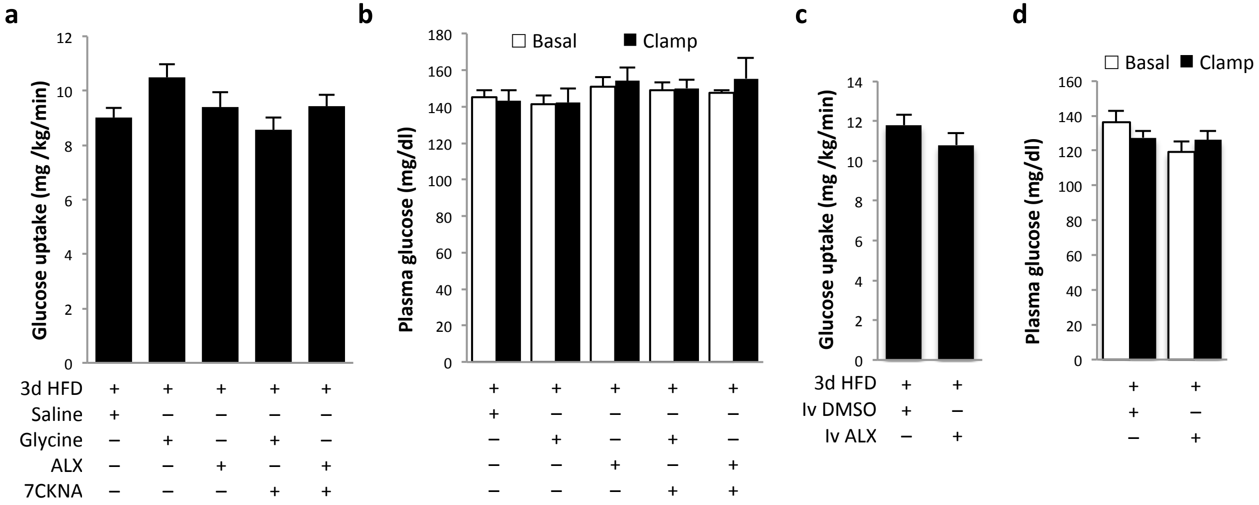

Supplementary Figure 5. Metabolic effects of the DVC and iv infusion of ALX in 3d-HFD rats.
(a) Glucose uptake and (**b**) basal and clamp plasma glucose levels during clamps with DVC infusion of saline (n=5), glycine (n=6), ALX (n=5), glycine+7CKNA (n=5), or ALX+7CKNA (n=5) in 3-d HFD rats, **(c)** Glucose uptake and (**d**) basal and clamp plasma glucose levels during clamps with iv infusion of 6% DMSO (n=7) or ALX (n=7) in 3-d HFD rats. Data are shown as the mean + SEM.

Supplementary Fig. 6

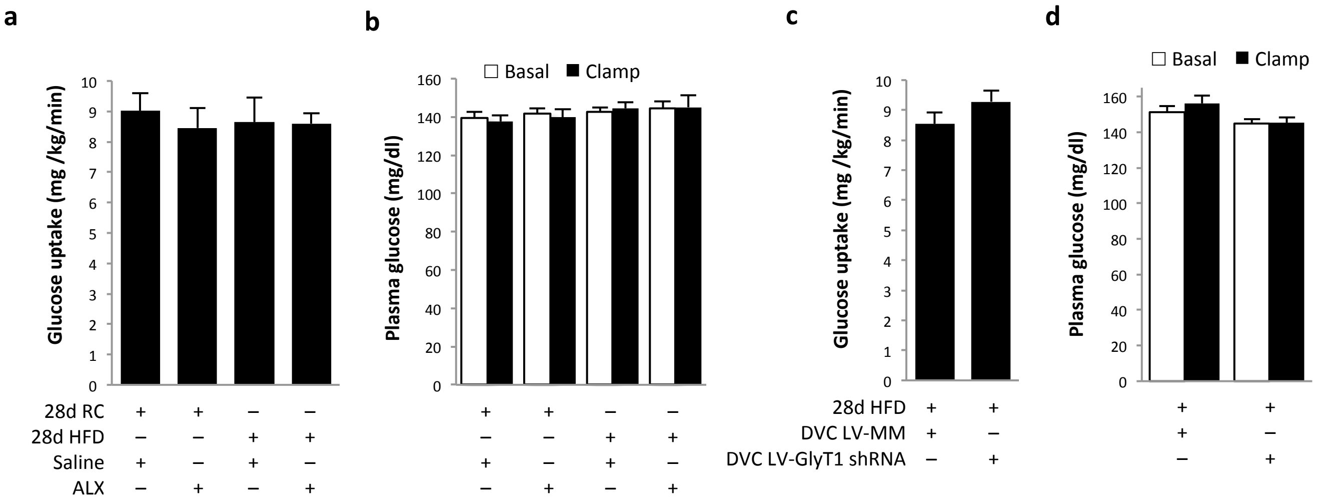

Supplementary Figure 6. Metabolic effects of chemical and molecular inhibition of DVC GlyT1 in obese rats.
(a) Glucose uptake and **(b)** basal and clamp plasma glucose levels during clamps in 28d RC-fed rats with DVC infusion of saline (n=5) or ALX (n=5) and in 28d HFD-fed rats with DVC infusion of saline (n=7) or ALX (n=7), **(c)** Glucose uptake and **(d)** basal and clamp plasma glucose levels during clamps in 28d HFD-fed rats injected with DVC LV-GlyT1 shRNA (n=10) or LV-MM (n=9). Data are shown as the mean + SEM.

Supplementary Fig. 7

a

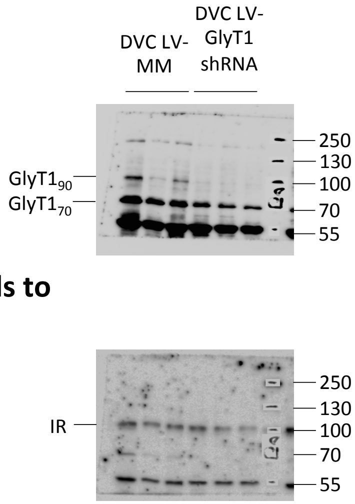

Corresponds to Fig. 2a

b

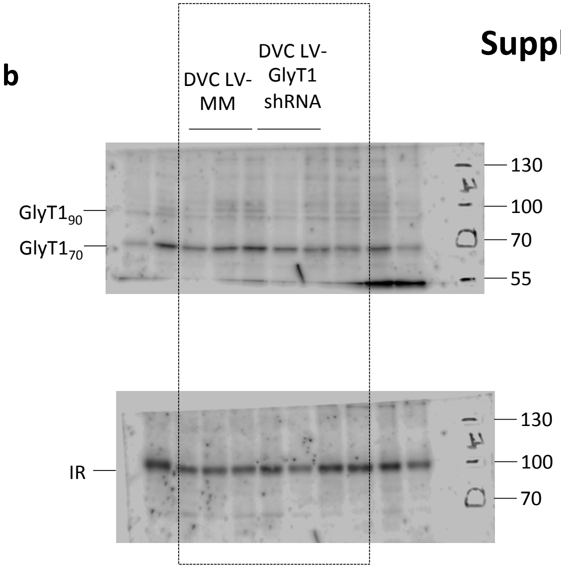

Corresponds to Fig. 2b

c

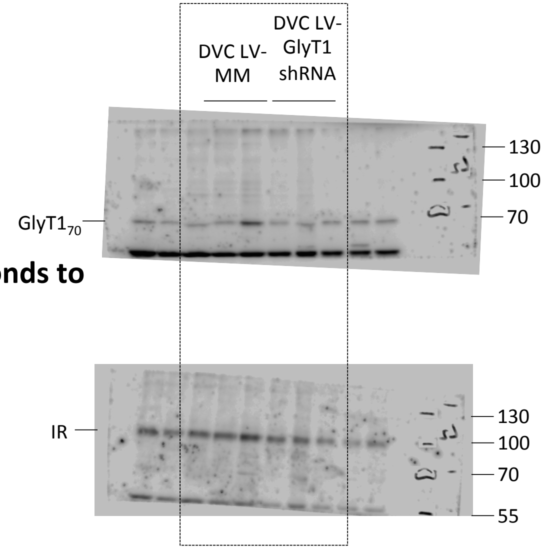

Corresponds to Fig. 2c

d

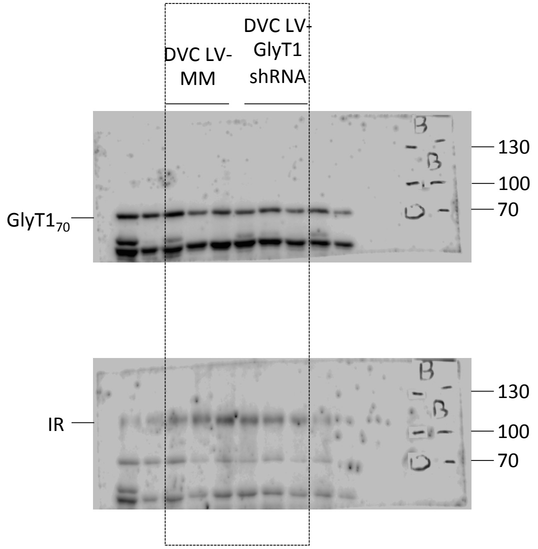

Corresponds to Fig. 2d

Supplementary Figure 7. Scans of uncropped Western blots.
Corresponding to representative images shown in **(a)**: Fig. 2a; **(b)**: Fig. 2b; **(c)**: Fig. 2c; **(d)**: Fig. 2d
